# Leveraging Ferroelectret Nanogenerators for Acoustic Applications

**DOI:** 10.3390/mi14122145

**Published:** 2023-11-23

**Authors:** Ziling Song, Xianfa Cai, Yiqin Wang, Wenyu Yang, Wei Li

**Affiliations:** 1 College of Integrated Circuit Science and Engineering, Nanjing University of Posts and Telecommunications, 9 Wenyuan Rd., Nanjing 210046, China; jxndsongziling@outlook.com (Z.S.); xianfacai@njupt.edu.cn (X.C.); yiqinwang99@163.com (Y.W.); 2 School of Mechanical Science and Technology, Huazhong University of Science and Technology, 1037 Luoyu Rd., Wuhan 430074, China; mewyang@hust.edu.cn; 3 Department of Mechanical Engineering, University of Vermont, 33 Colchester Ave., Burlington, VT 05405, USA

**Keywords:** ferroelectret nanogenerator, acoustic sensing, actuation, acoustic energy harvesting, ultrasound localization, ultrasonic medical imaging, nondestructive testing

## Abstract

Ferroelectret nanogenerator (FENG), renowned for its remarkable electromechanical conversion efficiency and low Young’s modulus, has gained significant attention in various acoustic applications. The increasing interest is attributed to the crucial role acoustic devices play in our daily lives. This paper provides a comprehensive review of the advancements made in using FENG for acoustic applications. It elaborates on the operational mechanism of FENG in acoustics, with a special focus on comparing the influence of different fabrication materials and techniques on its properties. This review categorizes acoustic applications of FENG into three primary areas: acoustic sensing, acoustic actuation, and acoustic energy harvesting. The detailed descriptions of FENG’s implementations in these areas are provided, and potential directions and challenges for further development are outlined. By demonstrating the wide range of potential applications for FENG, it is shown that FENG can be adapted to meet different individual needs.

## 1. Introduction

Acoustics, an interdisciplinary field that studies all forms of linear and nonlinear acoustic phenomena [1,2], ranges from micro to macro scales [3,4] and from infrasound to ultrasound [5,6,7]. With a strong crossover potential, acoustics overlaps with most modern scientific and technological disciplines, forming unique interdisciplinary directions such as medical ultrasound and ultrasonic human–computer interaction [8,9]. Driven by the demand for various acoustic devices integral to our daily lives, the field of acoustics has seen significant advancements in recent years. These devices cover a broad spectrum of applications, including object detection [10], space imaging [11], loudspeakers [12], and hydrophones [13,14]. 

To cater to the evolving needs of these applications, researchers are exploring new materials and technologies to enhance the performance and efficiency of acoustic devices. The traditional triboelectric nanogenerator (TENG) is widely used in acoustics, but its high internal impedance limits energy transfer efficiency [15,16]. A promising alternative is the FENG, a type of porous polymer with electrically charged internal cavities. FENG, with its exceptional electromechanical conversion efficiency and low Young’s modulus [17,18,19,20,21], allows for efficient bidirectional conversion between acoustic and electrical energy, offering more possibilities for the advancement of acoustics [22,23]. 

**Figure 1:**
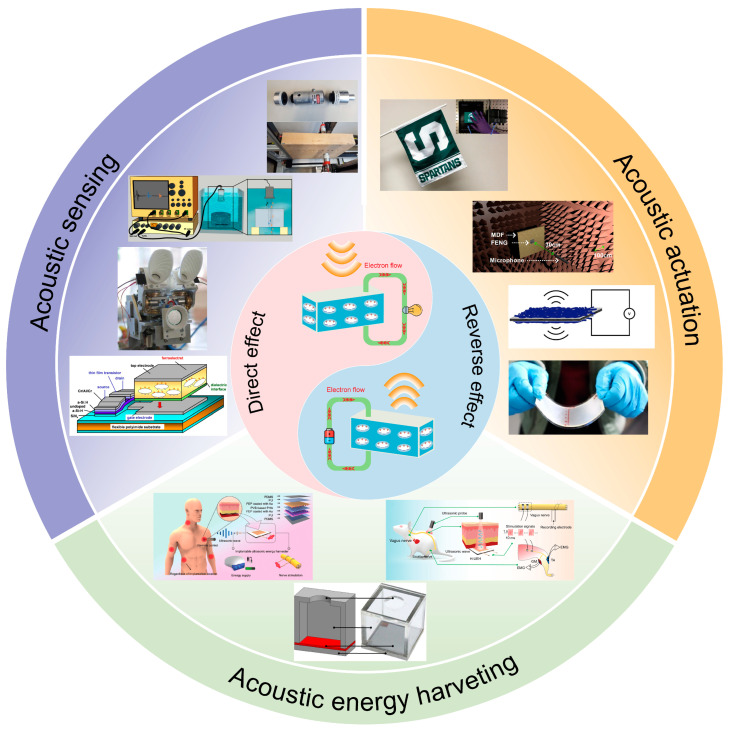
Categories defined for the acoustic applications of FENG. Reprinted with permission from Ref. [30]. Copyright 2006, American Institute of Physics. Reprinted with permission from Ref. [31]. Copyright 2011, IEEE. Reprinted with permission from Ref. [32]. Copyright 2020, Balkan Society of Geometers. Reprinted with permission from Ref. [33]. Copyright 2018, Springer. Reprinted with permission from Ref. [22]. Copyright 2017, Springer Nature. Reproduced with permission from Ref [34]. Copyright 2020, Academic Press Inc. Reprinted with permission from Ref. [35]. Copyright 2022, Elsevier. Reproduced with permission from Ref [36]. Copyright 2020, J. Acoustical Society of America. Reprinted with permission from Ref. [37]. Copyright 2019, IOP Publishing Ltd. Reprinted with permission from Ref. [16]. Copyright 2022, Wiley-VCH Verlag.

To date, FENGs have enabled intriguing advances in energy harvesting [24,25], robotics [26], biomechanical monitoring [27], etc. Moreira et al. [19] provided a brief introduction to FENG. Qiu et al. [28] introduced FENG applications in energy harvesting and air-coupled ultrasonic detection. Li et al. [29] reported the latest advancements in bioengineering systems based on FENG. However, the acoustic application of FENG has not been systematically introduced. To address this problem, leveraging FENG for acoustic applications has been reviewed in our work. This review introduces principles, theory with formula, design, fabrication, and characterization of FENG and their acoustic applications. We categorize the applications of FENG in acoustics into three main areas: acoustic sensing, acoustic actuation, and acoustic energy harvesting. Each of these areas illustrates the potential of FENG to enhance acoustic devices and systems. A comprehensive overview of FENG applications in acoustics is illustrated in Figure 1. The integration of FENG technology into acoustics holds great promise for transforming how we perceive, interact with, and harness sound in our everyday lives. 

## 2. Fundamentals of FENG

The term FENG refers to a type of cellular polymer that exhibits electret characteristics, hysteresis features akin to ferroelectric materials, and a quasi-piezoelectric effect post polarization [17,38,39,40]. The piezoelectric effect, discovered by French physicists Pierre and Jacques Curie in 1880, reveals that some crystals generate electric charges when subjected to changes in pressure or temperature [41,42,43,44]. The flexibility and high piezoelectric conversion efficiency of FENG have attracted significant research interest [22,45]. Unlike traditional piezoelectric materials with spontaneous electrical polarization, FENG is non-polar unless its internal void is charged by a dielectric-blocking microdischarge [17]. FENG’s charged cell structure amalgamates high piezoelectricity found in inorganic piezoelectric compounds (such as PZT) with the flexible thin film structure of organic piezoelectric polymers (like polyvinylidene fluoride (PVDF)) [29,46]. The unique cellular features of FENG enhance its sensitivity to stress and capability to store charge efficiently [29]. Thus, FENG holds substantial potential for acoustic applications. 

### 2.1. Working Principle of FENG

**Figure 2:**
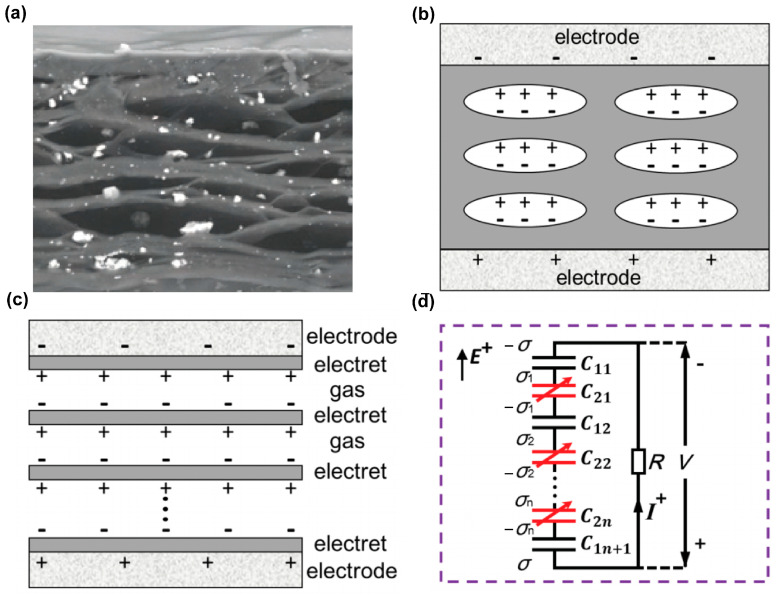
(**a**) Cross-sectional SEM image of a FENG based on cellular polypropylene. Reprinted with permission from Ref [17]. Copyright 2016, Elsevier BV. (**b**) Schematic illustration, (**c**) simplified structure, and (**d**) equivalent circuit model of a FENG. Reprinted with permission from Ref. [47]. Copyright 2016, John Wiley and Sons.

As a novel device, FENG has demonstrated its significance in bidirectional energy conversion between the electrical and mechanical fields. Many theoretical models have been established to study the mechanism of its macroscopic action [47,48,49]. Sessler et al. [48] developed an early model, which is widely used and consists of plane parallel solid layers and air layers. After that Li et al. [47] proposed an influential model as shown in Figure 2. A typical cross-sectional scanning electron microscopy (SEM) image of a cellular PP film is presented in Figure 2a. Solid electret layers alternate with gas gaps, with two electrodes positioned on the outermost top and bottom layers. The corresponding schematic diagram of a charged cellular electret is shown in Figure 2b. A simplified structure, such as that shown in Figure 2c, is considered, and Figure 2d illustrates the corresponding equivalent circuit model, in which the solid layers and gas bubbles are represented by fixed capacitors *C*_1*i*_ and variable capacitors *C*_2*j*_, and the thickness of the electret layer and the gas layer can be denoted by *d*_1*i*_ and *d*_2*j*_, respectively, with *i* = 1, 2, … *n* + 1 and *j* = 1, 2, … *n*, where *n* is the total number of bubble layers. 

After polarization, the opposite charges are captured on the solid–gas interfaces, forming dipole-like charge pairs. Influenced by the inner dipole-like charge pairs, charges are induced on the top and bottom electrodes with a negative charge density of −σ on the top and a positive charge density of σ on the bottom. 

Then, total quantity of charge *Q*(*t*) can be obtained as follows [47]:

(1) $$\begin{array}{l} Q(t)=Q_{0}\left(\frac{\varepsilon_{g}d_{e}}{\varepsilon_{r}d_{gas\_0}}+1\right)-Q_{0}\frac{\varepsilon_{g}d_{e}}{\varepsilon_{r}d_{gas\_0}}e^{-\frac{1}{RS\varepsilon_{0}}\left[\frac{d_{e}}{\varepsilon_{r}}t+\int_{0}^{t}\frac{d_{gas}(m)}{\varepsilon_{g}}dm\right]}\\ -Q_{0}\left(\frac{\varepsilon_{g}d_{e}}{\varepsilon_{r}d_{gas\_0}}+1\right)\frac{d_{e}}{RS\varepsilon_{0}\varepsilon_{r}}e^{-\frac{1}{RS\varepsilon_{0}}\left[\frac{d_{e}}{\varepsilon_{r}}t+\int_{0}^{t}\frac{d_{gas}(m)}{\varepsilon_{g}}dm\right]}\\ \times \int_{0}^{t}e^{\frac{1}{RS\varepsilon_{0}}\left[\frac{d_{e}}{\varepsilon_{r}}t+\int_{0}^{m}\frac{d_{gas}(x)}{\varepsilon_{g}}dx\right]}dm \end{array}$$

where $\varepsilon_{0}$ is the vacuum permittivity (~8.85 × 10^−12^ F/m), $\varepsilon_{r}$ and $\varepsilon_{g}$ are the relative permittivities of the electret material and the gas, respectively. The total electret thickness $d_{e}=\Sigma_{i=1}^{n+1}d_{1i}$ and the total thickness of gas layers $d_{gas}=\Sigma_{j=1}^{n}d_{2j}=n\bar{d_{2}}$, assuming that each gas gap thickness unit was fabricated with equal thickness ($\bar{d_{2}}$). And $d_{gas\_0}$ represents the thickness of the gas layer in its initial state.

Load output current and voltage can be derived as follows [47]:

(2) $$\begin{array}{l} I(t)=Q_{0}\left(\frac{\varepsilon_{g}d_{e}}{\varepsilon_{r}d_{gas\_0}}+1\right)\frac{d_{e}}{RS\varepsilon_{0}\varepsilon_{r}}-Q_{0}\frac{\varepsilon_{g}d_{e}}{RS\varepsilon_{0}\varepsilon_{r}d_{gas\_0}}\left[\frac{d_{e}}{\varepsilon_{r}}+\frac{d_{gas}(t)}{\varepsilon_{g}}\right]\\ \times e^{-\frac{1}{RS\varepsilon_{0}}\left[\frac{d_{e}}{\varepsilon_{r}}t+\int_{0}^{t}\frac{d_{gas}(m)}{\varepsilon_{g}}dm\right]}-Q_{0}\left(\frac{\varepsilon_{g}d_{e}}{\varepsilon_{r}d_{gas\_0}}+1\right)\frac{d_{e}}{R^{2}S^{2}\varepsilon_{0}^{2}\varepsilon_{r}}\\ \times \left[\frac{d_{e}}{\varepsilon_{r}}+\frac{d_{gas}(t)}{\varepsilon_{g}}\right]e^{-\frac{1}{RS\varepsilon_{0}}\left[\frac{d_{e}}{\varepsilon_{r}}t+\int_{0}^{t}\frac{d_{gas}(m)}{\varepsilon_{g}}dm\right]}\\ \times \int_{0}^{t}e^{\frac{1}{RS\varepsilon_{0}}\left[\frac{d_{e}}{\varepsilon_{r}}t+\int_{0}^{m}\frac{d_{gas}(x)}{\varepsilon_{g}}dx\right]}dm \end{array}$$

(3) $$\begin{array}{l} V(t)=Q_{0}\left(\frac{\varepsilon_{g}d_{e}}{\varepsilon_{r}d_{gas\_0}}+1\right)\frac{d_{e}}{S\varepsilon_{0}\varepsilon_{r}}-Q_{0}\frac{\varepsilon_{g}d_{e}}{S\varepsilon_{0}\varepsilon_{r}d_{gas\_0}}\left[\frac{d_{e}}{\varepsilon_{r}}+\frac{d_{gas}(t)}{\varepsilon_{g}}\right]\\ \times e^{-\frac{1}{RS\varepsilon_{0}}\left[\frac{d_{e}}{\varepsilon_{r}}t+\int_{0}^{t}\frac{d_{gas}(m)}{\varepsilon_{g}}dm\right]}-Q_{0}\left(\frac{\varepsilon_{g}d_{e}}{\varepsilon_{r}d_{gas\_0}}+1\right)\frac{d_{e}}{RS^{2}\varepsilon_{0}^{2}\varepsilon_{r}}\\ \times \left[\frac{d_{e}}{\varepsilon_{r}}+\frac{d_{gas}(t)}{\varepsilon_{g}}\right]e^{-\frac{1}{RS\varepsilon_{0}}\left[\frac{d_{e}}{\varepsilon_{r}}t+\int_{0}^{t}\frac{d_{gas}(m)}{\varepsilon_{g}}dm\right]}\\ \times \int_{0}^{t}e^{\frac{1}{RS\varepsilon_{0}}\left[\frac{d_{e}}{\varepsilon_{r}}t+\int_{0}^{m}\frac{d_{gas}(x)}{\varepsilon_{g}}dx\right]}dm \end{array}$$

From Equations (1)–(3), it is apparent that the generated outputs are determined using the material parameters ($\varepsilon_{g}$, $\varepsilon_{r}$, $d_{e}$, and $d_{gas\_0}$), the amount of charge after polarization, ($Q_{0}$), and the stimulus mode ($d_{gas}(t)$ as well as the external load (*R*)). Moreover, a large relative permittivity, a high polarization, a matched external load, and strong stimulation are beneficial for enhancing the output [47,50].

**Figure 3:**
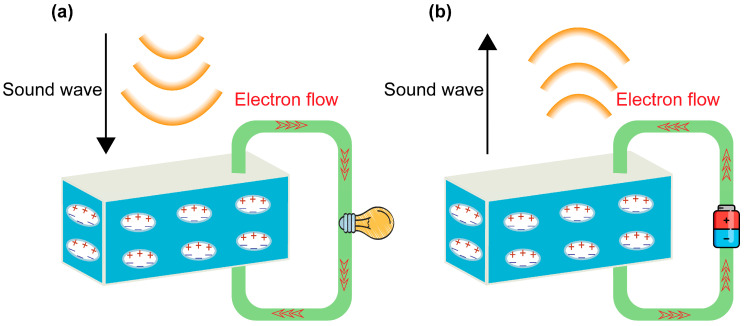
(**a**) The mechanism of using FENG to convert sound energy into electrical energy. (**b**) The mechanism of using FENG to convert electrical energy into sound energy.

The piezoelectric effect in FENG stems from changes in engineered macroscopic electric dipoles to generate displacement currents (and vice versa) [29]. Within FENG, engineered macroscale voids with opposite polarity charges trapped on top and bottom surfaces are uniformly distributed, forming giant permanent dipoles [51]. The mechanism of this energy transformation is depicted in Figure 3a,b. When subjected to mechanical stress or strain, such as vibrations or sound waves, the embedded voids within the FENG film undergo deformation. This deformation prompts the redistribution of electric charges and changes in the dipole moment, leading to the generation of an electric potential difference across the film [22,51,52,53]. This potential difference can be harvested, used to power electronic devices, or stored in energy storage systems. Conversely, if additional charges are transferred to surface electrodes, the alteration in the charge density on these electrodes reshapes the giant dipoles within the FENG, exhibiting a reverse electromechanical interaction effect [22]. The energy conversion efficiency in FENG depends on several factors such as the piezoelectric properties of the ferroelectret material, the applied mechanical deformation, and the FENG device design [51]. Utilizing the positive and inverse piezoelectric effects in FENG enables the bidirectional conversion of acoustic and electrical energy. The energy conversion mechanism of FENG paves the way for the development of acoustic applications.

### 2.2. Preparation Method of FENG

**Figure 4:**
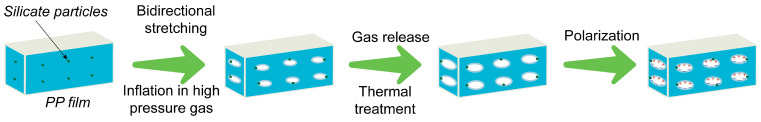
A schematic description of the processing steps of FENG.

Common processing materials for FENG include polypropylene (PP) [17,54,55], irradiated cross-linked polypropylene (IXPP) [36], and expanded polytetrafluoroethylene (ePTFE) [56,57,58,59,60]. These materials offer excellent flexibility and processing potential, allowing them to be manufactured as thin cellular films. A typical method of preparing FENG using high-pressure gas injection combined with small inorganic particles is shown in Figure 4, using PP film containing tiny foreign silicate particles [17,61,62]. When the film is stretched in two perpendicular directions, these inorganic particles act as stress concentrators or microcracks, resulting in lens-shaped voids in the selected materials. Concurrently, high-pressure gas diffuses into the void-filled film, equalizing the internal and external pressures. The external gas pressure is then abruptly released, causing a dramatic swelling of the voids in the FENG film. To stabilize and stiffen the swelling voids at room temperature, thermal treatment is performed to increase the polymer matrix’s crystallinity. After the structure is thermally stabilized, a high electric field is applied to break down the gas molecules into plasma, depositing opposite charges on the upper and lower surfaces of each void [63]. Subsequently, a metal layer can be affixed to both sides of the film using several methods such as spin-coating conductive materials and applying conductive tape [17,64].

Manufacturing techniques like the foaming process, sandwich layer structure, and micro-patterning method are also broadly applied to process FENG [65,66]. Some of these methods allow the inner cavities of FENG to be geometrically controlled, thereby changing the piezoelectric coefficient [19]. The main charging methods of FENG include corona charging, contact charging, soft X-ray irradiation, and electron beam injection [18,66]. By applying a high voltage, the cavities of polymer foams are charged and generate tiny dielectric barrier microdischarges within the cavities [67]. Table 1 summarizes the common applications, manufacturing, and charging methods of FENG. It is noteworthy that FENGs exhibiting a high piezoelectric coefficient hold significant promise for a wide range of applications [68].

Proper selection of material and charging methods can yield high-quality FENG. For example, using high-pressure gas injection combined with small inorganic particles, a high piezoelectric coefficient can be achieved for PP polymer [17]. For ePTFE, the method of FEP-ePTFE-FEP-ePTFE-FEP stack can achieve excellent performance.

Achieving a high piezoelectric coefficient in FENGs can be accomplished through improvements in both the manufacturing and charging methods. The introduction of parallel controlled arrays during FENG fabrication has been shown to yield abnormally high piezoelectric coefficients [16]. Air breakdown may occur at the parallel controlled arrays due to the strong electric field formed by a pair of electrets with opposite electric charges. Therefore, such a dielectric layer with low relative permittivity can enhance the piezo-response in the electret/dielectric/electret sandwich-shaped systems, and this design enables enhanced strain to occur in the electret layer [16,75]. Additionally, prefilling bipolar ions before charging FENGs has proven to be an effective technique for enhancing the piezoelectric coefficient [46]. 

The piezoelectric reliability and durability of FENG are crucial aspects of its performance. Early flexible FENGs were predominantly fabricated using polypropylene (PP), which, unfortunately, exhibited poor thermal stability and durability [68]. In contrast, laminated FENGs, constructed from polytetrafluoroethylene (PTFE) and/or fluorinated ethylene propylene (FEP) films, demonstrated improved thermal stability and durability, effectively addressing this limitation [68,72]. 

Since the piezoelectric properties of the ferroelectret originate from charged pores, it is of interest to evaluate their surface potential decay with time [74]. A considerable amount of degradation in the piezoelectric coefficient can be seen over time [29,76]. Assagra et al. [70] fabricated 3D-printed PP films with a piezoelectric coefficient *d*_33_ of almost 200 pC/N but also showed a significant decay of the FENG over the first 20 days after charging with an average loss of 40% of the initial piezoelectricity. The high charge-capturing ability and low elastic modulus of the porous polymer bring about problems with a loss of positive and negative charges on the internal surfaces of the artificial voids inside the FENG, which have been considered the main reasons for the stabilization of the piezoelectric response [29,74]. Utilizing extra processing techniques, such as charging and pre-aging at high temperatures, may improve performance over the long run [60,77]. Further research into novel materials and micro/nanostructures on the void’s inner surface may also shed light on how to stabilize FENG performance over time. Shi et al. [69] manufactured PET silk ferroelectret with an initial piezoelectric coefficient *d*_33_ of about 1650 pC/N and it retained around 97% of its initial piezoelectric coefficient *d*_33_ over 30 days. The effect of environmental elements on FENG performance, such as humidity and temperature, must also be taken into account. Therefore, another tactic to help maintain residual charges and guarantee stability is proper encapsulation [29].

## 3. Acoustic Applications of FENG

### 3.1. Acoustic Sensing

Acoustic sensing transforms sound signals from an external sound field into electrical signals. The usual acoustic sensing devices include microphones, hydrophones, and ultrasonic transducers, typically used for non-destructive testing, ultrasonic locating, and other purposes [78,79]. Compared to traditional piezoelectric polymers, FENG exhibits high piezoelectricity following proper electrical charging, hence their suggested use in acoustic sensing [80]. Moreover, FENG acoustic sensing takes advantage of low cost and excellent electroacoustic characteristics, making it attract diverse studies in communication, noise control, and environmental monitoring [15,81]. Table 2 summarizes common applications of FENG devices in acoustic sensing, including microphones, ultrasonic localization, ultrasonic medical imaging, and nondestructive testing [78,82,83]. To date, FENG has proven efficient, economical, lightweight, reliable, and compatible with acoustic sensing across a wide frequency range [29].

#### 3.1.1. Microphones

**Figure 5:**
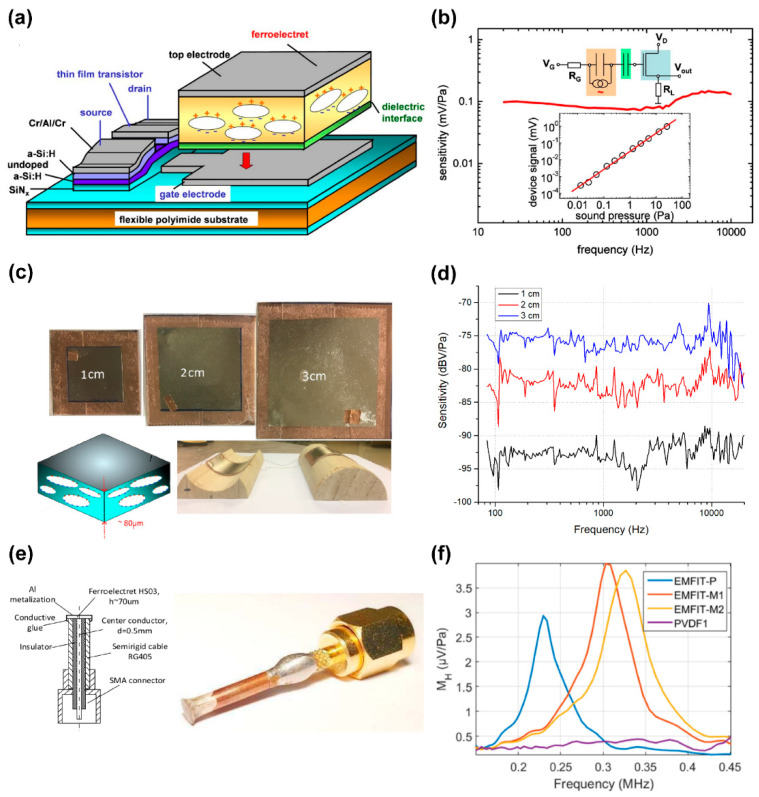
(**a**) Sketch of the transducer arrangement for the investigation of the ferroelectret field effect and. (**b**) Microphone operation of the ferroelectret thin-film field-effect transistor with V_GS_ = 8 V and V_DS_ = 8 V. Reprinted with permission from Ref. [30]. Copyright 2006, American Institute of Physics. (**c**) Samples with different areas; the inset shows a cross-section diagram of FENG and (**d**) the frequency response of 3 samples. Reprinted with permission from Ref. [85]. Copyright 2019, IEEE. (**e**) Microphone construction (left) the assembled device (right), and (**f**) microphone ac response obtained by three-transducer calibration. Reprinted with permission from Ref. [87]. Copyright 2022, IEEE.

Microphones are a kind of common acoustic sensing application that is widely used in our lives. The microphones prepared with FENG are simple to make, low in cost, and have attracted wide attention [80]. However, they also have problems such as low signal-to-noise ratio, power noise interference, poor sensitivity, and insufficient dipole density of FENG microphones [85,86]. As early as 2005, Hillenbran et al. [80] developed an inexpensive, easily constructed microphone, utilizing five stacked PP films, displaying a sensitivity of approximately 10.5 mV/Pa at 1 kHz. Later in 2006, Graz et al. [30] introduced a ferroelectret field-effect transistor, integrated with a field-effect transistor, functioning as a microphone (see Figure 5a). Figure 5b displays the excellent linearity of the ferroelectret field-effect transistor’s microphone response with V_GS_ = 8 V and V_DS_ = 8 V. Dsouza et al. [85] investigated the influence of FENG parameters on microphone performance, producing flexible FENG-based microphones of varying shapes and areas (shown in Figure 5c). Figure 5d displays the frequency response of three kinds of FENG-based microphones with differing areas, revealing reconfigurable features because their sensitivity and directivity change with area and frequency. Li et al. [22] confirmed the performance of FENG-based microphones using high-fidelity music recording and voice recognition security systems. They expanded FENG applications by cleverly using its bi-directional energy conversion characteristics to fashion a simple, light, flexible, wearable microphone. 

Moreover, studies have been conducted on acoustic sensing in the ultrasound field. Svilainis et al. [87] reported on the design and performance evaluation of a miniaturized air-coupled ultrasonic microphone made of FENG film. The construction of the microphone (left) and the assembled device (right) are shown in Figure 5e. They evaluated the sensitive area size and AC response of the microphone using laser ultrasound and three-transducer reciprocity calibration techniques, respectively. The AC response is shown in Figure 5f. The results showed that the microphone had high sensitivity near its thickness resonance frequency with a 0.5-mm sensitive element size, suitable for measuring ultrasonic fields in air. Compared with the PVDF microphone of the same structure, the FENG-based microphone was less expensive and easier to fabricate. Zhang et al. [84] fabricated microphones with excellent performance using FENG films and measured their frequency response in the range of 0.1~100 kHz. Their results revealed that the microphone had high sensitivity and broadband characteristics but was influenced by thickness resonance and diffraction effects. They also studied the effect of annealing temperature on microphone sensitivity and dynamic piezoelectric coefficient, finding that after 300 min of annealing at 125 °C, the piezoelectric coefficient reduced to about 40% of the initial value.

#### 3.1.2. Ultrasonic Localization

Ultrasonic localization, a technology that utilizes transmitted ultrasonic signals and received echo signals to determine an object’s position and distance, finds extensive use in fields such as medicine, industry, navigation, and robotics [88]. 

**Figure 6:**
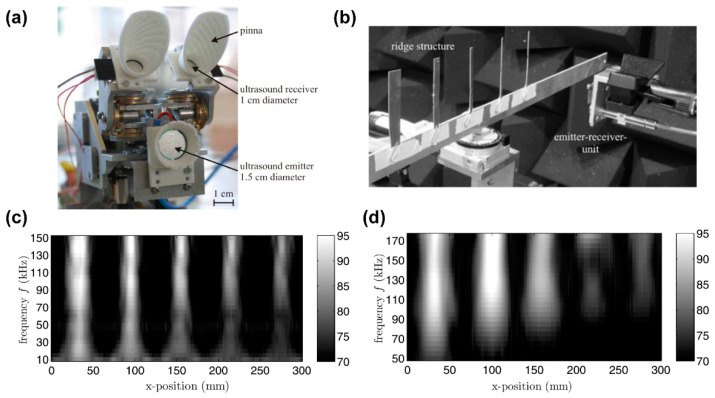
(**a**) Photograph of the artificial bat head, which mainly consists of an ultrasound emitter and two ultrasound receivers. Each pinna can be rotated by an electric motor. (**b**) Measurement setup for the determination of the lateral spatial resolution. A 3D translation unit is used to vary the distances between the ridge structure and the emitter–receiver-unit, (**c**) Waterfall diagrams (excitation frequency f over x-position; lateral spacing 1 mm; frequency increment 5 kHz) of the reflected SPL (in dB) for the ridge structure consisting of five forks with different lateral dimensions and 5 cm axial distance between the emitter–receiver unit and the ridge structure and (**d**) 20 cm axial distance between the emitter–receiver unit and the ridge structure. Reprinted with permission from Ref. [31]. Copyright 2011, IEEE.

The development of air-coupled ultrasonic transducers with high efficiency and high sensitivity is possible using FENG, offering significant implications for ultrasonic imaging, localization, non-destructive testing, and more [89,90]. The location function may be actualized using artificial technology, imitating the biological ultrasonic localization system of bats [91]. In 2005, Streicher et al. [82] manufactured sensors capable of transmitting and receiving broadband ultrasonic signals ranging from 20~200 kHz, which are used to emulate bats’ echolocation systems. These sensors employed thickness oscillators using EMFi sheets of varying sizes and layers to act as transmitters and receivers. Subsequently, in 2011, Rupitsch et al. [31] proposed an ultrasonic transducer based on FENG with considerable bandwidth and a high piezoelectric strain constant. They fabricated single-element transducers and array transducers of different shapes and sizes using this transducer, testing and simulating their transmitting and receiving characteristics. Notably, they demonstrated artificial bat heads, based on FENG ultrasonic transducers, for ultrasound localization using a biometric sonar system mimicking a bat. As depicted in Figure 6a, the setup primarily consists of an ultrasound emitter (1.5 cm diameter) and two ultrasound receivers (1.0 cm diameter). These three transducers were crafted from a single-layered FENG. Employing a pinna that can be rotated by an electric motor, the reflected sound waves are concentrated. Figure 6b illustrates the measurement setup located in the anechoic room, where a 3D translation unit traverses the rigid structure to the emitter–receiver unit. Figure 6c,d display measurement results for varying x-positions and different axial distances (5 cm and 20 cm) between the emitter–receiver unit and the rigid structure. With the ultrasonic localization function, more applications are anticipated.

#### 3.1.3. Ultrasonic Medical Imaging

Ultrasonic medical imaging is a non-invasive, relatively straightforward, swift, and real-time imaging technology that uses ultrasound to generate images of tissues and organs within the human body. It is routinely utilized for diagnostic, monitoring, and treatment guidance purposes [92]. Pulse echo technology is predominantly used in ultrasonic medical imaging, and the spectral analysis of reflected pulses can disclose the internal structure of scatterers and various organs in medical imaging [32,93,94]. Ultrasonic transducers with a concise impulse response and a broad frequency band response are desirable for ultrasonic imaging and quantitative echography [32]. 

**Figure 7:**
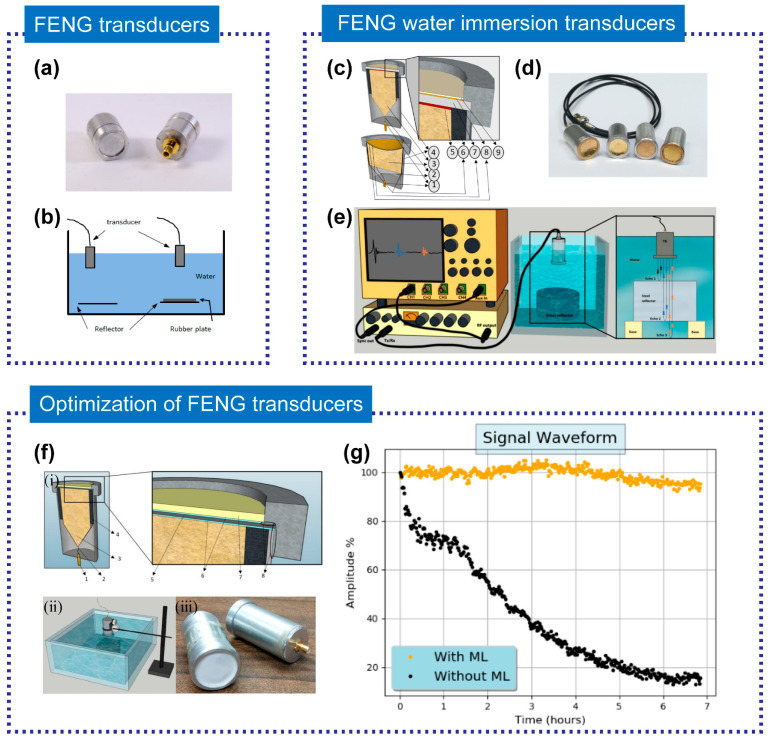
(**a**) Picture of the two 10 mm diameter prototypes of water immersion transducers using FF film (left: front face, right: rear face with SMB connector), and (**b**) schematic representation of the experimental set-up. Reprinted with permission from Ref. [93]. Copyright 2016, IEEE. (**c**) Transducer design for flat and spherically focused prototypes. (**d**) Picture of transducer prototypes, from left to right: HS03 focused (radius of curvature: 22 mm), HS03 focused (radius of curvature: 35 mm), HS06 flat, HS03 flat, and (**e**) experimental set-up for pulse-echo measurements. Reprinted with permission from Ref. [32]. Copyright 2020, Balkan Society of Geometers. (**f**) The schematic of FENG transduces design and test: (i) Cross-section view of transducer design (left) and detail of the connections (right). (ii) Schematic representation of the experimental set-up for positioning the produced prototype transducer for water immersion pulse-echo characterization. (iii) The prototype of FENG transduces. (**g**) Variation with time of immersion of the normalized echo amplitude for two prototype transducers: Transducer with unprotected Al metallization and transducer with one matching layer. Reprinted with permission from Ref. [96]. Copyright 2019, IEEE.

The FENG device, with its low impedance and high electromechanical conversion efficiency, is ideal for ultrasonic medical imaging [95]. In 2016, Gómez et al. [93] explored the feasibility of using ferroelectret films to fabricate wide-band ultrasonic transducers for applications such as water immersion and medical imaging. They created and characterized ultrasonic transducers of various sizes. Figure 7a illustrates two 10 mm diameter prototypes of water immersion transducers using ferroelectret film, which were characterized in a pulse-echo operation mode during water immersion, as seen in Figure 7b. These transducers exhibited a broad bandwidth (0.3~2.5 MHz), though their sensitivity was low, necessitating further research or improvement efforts. The researchers also discovered that these transducers could be deployed for the detection and characterization of layered reflectors, like rubber sheets and steel plates, which have significant implications for medical imaging.

The FENG-based ultrasonic transducer can enhance spatial resolution and spectrum analysis capabilities in ultrasonic medical imaging. In 2020, Quirce et al. [32] reported the design, fabrication, and testing of water immersion pulse-echo ultrasonic flat and spherically focused transducers based on two different PP films (HS03 and HS06). The design sketches and prototype are shown in Figure 7c,d while the experimental setup is displayed in Figure 7e. They demonstrated that FENG films have a very wide bandwidth (0.2~2.7 MHz) and a short pulse duration (2~3 μs) in water, making them suitable for medical imaging. The FENG-based ultrasonic transducer can enhance spatial resolution and spectrum analysis capabilities in ultrasonic medical imaging. 

In addition, a quantity of related research focuses on how to improve the characteristics of FENGs for water-coupled ultrasonic examination. Aguilar et al. [96] studied how to adapt and optimize PP films as the primary piezoelectric element for water immersion ultrasonic transducers. They proposed a new transducer design that implemented a gold coating and epoxy resin filled with microspheres to protect the FE film’s metallization layer and enhance stability in water. They also improved impedance matching to the water, thereby preserving the bandwidth. The prototype and experimental setup for water immersion pulse-echo characterization are displayed in Figure 7f, and Figure 7g demonstrates the variation in normalized echo amplitude for different immersion times of the transducer in water. The results show that the electrodes of the transducer without protection will degrade quickly, whereas the transducer with a matching layer maintains better stability. Additionally, FENG can serve as a matching layer for ultrasonic transducers, further expanding its applications [97,98].

#### 3.1.4. Nondestructive Testing

Nondestructive testing (NDT) is a technique employed to evaluate internal defects or the performance of a material, component, or system. Unlike traditional destructive testing methods, NDT technology allows testing and evaluation without causing damage to the object being tested. The purpose is to guarantee the quality of materials and components and provide reliability and safety information. Therefore, it is utilized across various industries, including manufacturing, aerospace, automotive, energy, construction, and infrastructure [99,100]. Ultrasonic transducers made of FENG offer high sensitivity, broad frequency response, and high signal-to-noise ratio. Consequently, FENG is extensively used for testing defects in wood, fabric, and other materials [33,101,102,103].

**Figure 8:**
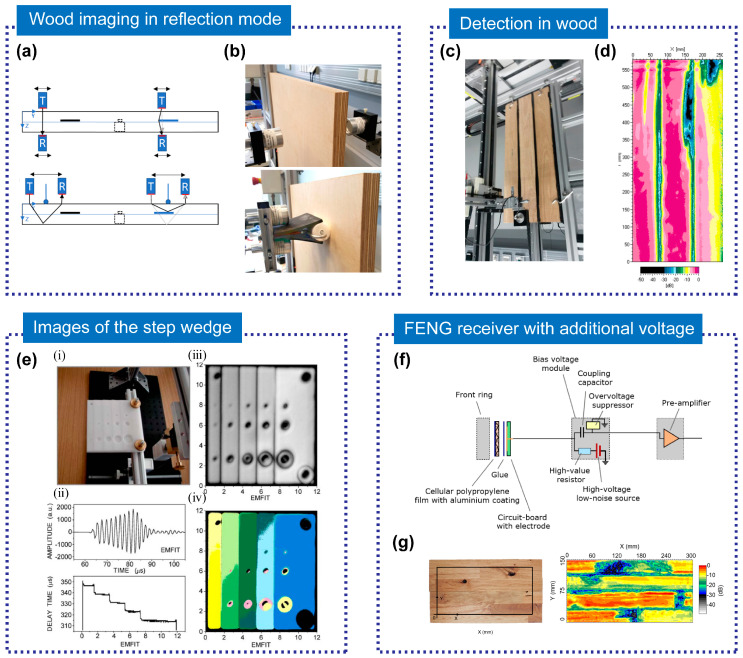
(**a**) Test configuration of the multiplex specimen for the back wall and defect detection utilizing transmission (up) and reflection (down) measurements and (**b**) experimental setup in transmission (up) and reflection with a foam roller (down). Reprinted with permission from Ref. [83]. Copyright 2020, Elsevier Ltd. (**c**) Scanning measurement system and (**d**) C-scan attenuation (dB) image from FENG. Reprinted with permission from Ref. [104]. Copyright 2020, Springer Berlin Heidelberg. (**e**) Non-contact ultrasonic imaging using EMFIT transducers (pulse-echo mode) (i) Tested polyethylene step wedge; (ii) typical shape of the pulse transmitted by FENG and example of the delay time profile along the *X*-axis; (iii) amplitude and (iv) delay time images of the step wedge, obtained by FENG transducers. Reprinted with permission from Ref. [105]. Copyright 2007, American Institute of Physics. (**f**) Receiver with a bias voltage module that adds external bias voltage to the existing internal voltage of the FENG and (**g**) glued laminated timber with an indicated area of inspection (left) and inspection results (right), using ferroelectret transducers with external bias voltage. Reprinted with permission from Ref. [106]. Copyright 2019, IEEE.

Using FENG for nondestructive testing of wood can be an efficient pathway to increase the acceptance of wooden structures and promote sustainability in civil engineering. However, the technique is affected by the anisotropy, surface waves, and layer structure of wood, which can cause signal attenuation and interference [83]. Vössing et al. [83] introduced a reflection method for detecting wood using an air-coupled ultrasonic transducer based on PP film. This method has a high signal-to-noise ratio and high resolution, and it can inspect wood in reflection mode. They carried out experiments on different wood samples and compared them with transmission mode. The test configuration and experimental setup are shown in Figure 8a,b. Their results showed that the reflection mode can accurately inspect defects like millings, cavities, and drillings, and determine the depth of the defects. 

The use of novel FENG transducers improved the sensitivity and signal-to-noise ratio of air-coupled ultrasound. Tiitta et al. [104] researched a method for detecting natural defects in wood using an air-coupled ultrasound technique. They used gas matrix piezoelectric and FENG transducers and applied additional bias voltage to the FENG receivers. As shown in Figure 8c,d, they compared the ultrasound responses with the visual detection results of the defects by scanning measurements and signal analysis. Their findings indicated that ultrasonic detection is reliable.

Transducers made of cellular PP are well-suited for air-coupled ultrasonic detection due to their extremely low Young’s modulus and low density, resulting in a favorable acoustic impedance for the transmission of ultrasonic waves between the transducer and air [33]. Bovtun et al. [105] studied the dielectric and electromechanical properties of commercially available cellular PP and the development of non-contact ultrasonic transducers based on them. They found that FENG films have low acoustic impedance, low dielectric and mechanical losses, and high-frequency electromechanical resonance, which makes them suitable for air-coupled ultrasonic applications. The prototype transducers, which are based on FENG films, exhibited high sensitivity and resolution in non-contact ultrasonic imaging and testing of a polyethylene step wedge with holes, as shown in Figure 8e. 

The signal-to-noise ratio of the FENG transducer can be improved by attaching an external voltage. In 2019, Gaal et al. [106] reported an air-coupled ultrasonic receiver based on charged cellular PP, which includes a high-voltage module providing additional DC bias voltage, as shown in Figure 8f. This bias voltage led to a 15 ± 1 dB increase in the signal-to-noise ratio of the receiver. The receiver, combined with a cellular PP transmitter, was successfully applied to the non-destructive testing of glued-laminated timber. It enabled imaging of the internal structure of these specimens, which had a thickness of approximately 4 cm, as shown in Figure 8g.

In addition, cellular PP transducer films are well-suitable for structural testing of flexible fabrics [107]. Pazos-Ospina et al. [108] reported a FENG-based air-coupled ultrasonic phased array for nondestructive testing of textiles. They adopted a dual-focalization scheme, using curved array elements to achieve natural focusing in the vertical direction and electronic focusing in the horizontal direction, thus obtaining a homogeneous spatial resolution on the inspection plane. This phased array has the advantages of low cost, simple fabrication, high flexibility, etc., achieving high resolution and high signal-to-noise ratio in textile testing. Moreover, FENG-based air-coupled ultrasonic transducers have demonstrated effective performance in nondestructive testing of metal adhesively bonded structures in the aerospace industry and lightweight structures [95,109].

### 3.2. Acoustic Actuation

An acoustic actuation is a device that converts electrical energy into sound vibrations. The high sensitivity and efficient electromechanical conversion properties of FENG make it ideal for acoustic actuation applications including loudspeakers, acoustic levitators, and vortex generators as summarized in Table 3 [35,36,110].

#### 3.2.1. Loudspeakers

**Figure 9:**
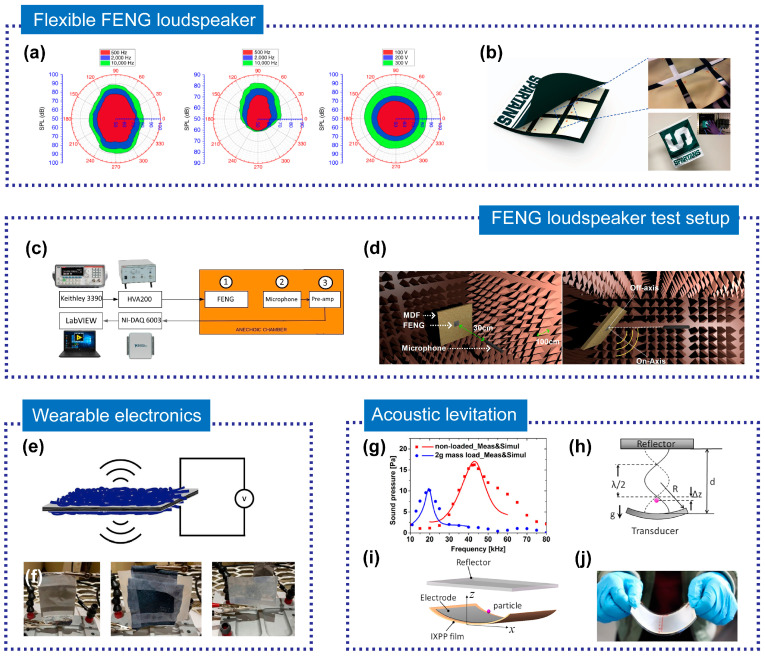
(**a**) SPL polar plots under different frequencies or voltage and (**b**) design and demonstration of the music-playing flag. Reprinted with permission from Ref. [22]. Copyright 2017, Springer Nature. (**c**) The schematic diagram of the test setup inside an anechoic chamber and (**d**) a schematic of SPL directivity measurement. Reproduced with permission from Ref. [34]. Copyright 2020, Academic Press Inc. (**e**) Schematic diagram of the working principle of a ferroelectret loudspeaker and (**f**) sputter-coated polypropylene ferroelectret loudspeaker, FEP-ePTFE-FEP-ePTFE-FEP ferroelectret with PEDOT: PSS-coated fabric loudspeaker, and PEDOT: PSS-coated polypropylene ferroelectret loudspeaker. Reprinted with permission from Ref. [35]. Copyright 2022, Elsevier. (**g**) Measured and simulated radiated sound pressure of non-loaded (red line) and 2 g loaded (blue line) IXPP films at a 10 cm distance. (**h**) Schematic of the mechanism of acoustic levitation for an acoustic levitator with a curved transducer, (**i**) 3D mode of an IXPP ferroelectret film-based acoustic levitator, and (**j**) a prototype of IXPP ferroelectret film-based levitator. Reproduced with permission from Ref [36]. Copyright 2020, J. Acoustical Society of America.

Loudspeakers, electronic devices that convert electrical signals into sound signals, are widely used [111,112]. Compact and lightweight FENG-based loudspeakers can be integrated into various devices and surfaces. Li et al. [22] introduced a flexible loudspeaker based on FENG and they demonstrated the sound pressure level (SPL) directivity of FENG as a loudspeaker in three different configurations (free-standing, substrate-held, and rolled-up) as shown in Figure 9a. The results showed that the rolled-up loudspeaker has the potential for omnidirectional sound production. Particularly, a music-playing flag incorporating FENG is illustrated in Figure 9b. Dsouza et al. [34] reported on a flexible loudspeaker based on FENG, measuring the SPL, linearity, directivity, folding effect, and other parameters of FENG. They established a theoretical model based on the Boundary Element Method (BEM) to explain and validate the acoustic characteristics of FENG, exploring its performance in the ultrasound range. The test setup schematic diagram is shown in Figure 9c,d. FENG-based loudspeakers could enhance human–computer interaction technology when used in wearable devices. Ploner et al. [35] developed, prototyped, and tested a novel ultrathin, all-organic, fabric-based ferroelectret loudspeaker for wearable electronics. The working principle schematic diagram is shown in Figure 9e. They fabricated loudspeakers with different types of electrodes and ferroelectrets and investigated their performance as shown in Figure 9f. The loudspeaker composed of FEP-ePTFE-FEP-ePTFE-FEP ferroelectret and PEDOT: PSS-coated fabric electrodes demonstrated good temperature stability, biocompatibility, and wearability in the frequency range of 5~15 kHz. 

#### 3.2.2. Acoustic Levitators

FENG has the advantages of flexibility, lightweight, broadband, simple structure, low cost, and environmentally friendliness, providing a novel and effective method for future acoustic suspension applications. Xue et al. [36] designed and fabricated a large-area, flexible broadband acoustic levitator based on irradiated cross-linked polypropylene (IXPP) film. They simulated and measured the radiated sound pressure of the fabricated FENG as shown in Figure 9g. The device utilized the high piezoelectric effect and the very low acoustic impedance of IXPP thin film to generate a strong acoustic radiation force in the air, achieving stable suspension and precise manipulation of various material particles. The mechanism of acoustic levitation for an acoustic levitator with a curved transducer is shown in Figure 9h, while Figure 9i,j show the 3D model of an IXPP ferroelectret film-based acoustic levitator and the prototype of the levitator, respectively. By adjusting the curvature radius of the thin film transducer and the distance from the reflector, the position of the suspended particles in space can be controlled. 

#### 3.2.3. Vortex Generators

FENG has significant potential in the generation of ultrasonic vortices, offering a new option for the use of ultrasonic vortices in future engineering applications. Ealo et al. [110] introduced a method for fabricating an air ultrasonic vortex generator using FENG, capable of producing sound waves with spiral wavefronts at ultrasonic frequencies in the air. Using a simple and low-cost fabrication process that involves pasting the FENG film onto a substrate on a tangential/helical surface, the FENG’s high mechanical flexibility and broad-band response enable special sound field customization. Through theoretical simulation and experimental measurement, it was verified that the fabricated generator can produce high-quality spiral wavefronts in different observation planes, compared with the ideal Gauss Laguerre beam.

### 3.3. Acoustic Energy Harvesting

Acoustic energy harvesting is an emerging field in green energy technology due to its ubiquitous nature. However, the utilization of acoustic energy presents a challenge due to the low power density in the sound field [113,114,115]. FENG with high piezoelectric properties and relatively low acoustic impedance are promising candidates for acoustic energy harvesting [56,116]. Table 4 summarizes the common applications of FENG devices in acoustic energy harvesting containing ambient sound energy harvesting and implantable electronics power supply. Ambient sound energy, such as that generated by mobile phone calls or environmental noise, is often wasted but has the potential to be converted into useful electrical energy [15,66]. Implantable electronic power supplies that scavenge wireless mechanical energy from ultrasound possess remarkable potential in advanced medical protocols for neuroprosthetics, wireless power, and biosensors [16]. As an energy harvester, FENG has the advantages of simple fabrication, excellent thermal stability, effective piezoelectric effect, and high output power, which could be used for wide-band and high-efficiency sound energy absorption [117,118]. Utilizing FENG could facilitate the reuse of ambient sound energy and promote the development of a wireless power supply.

**Figure 10:**
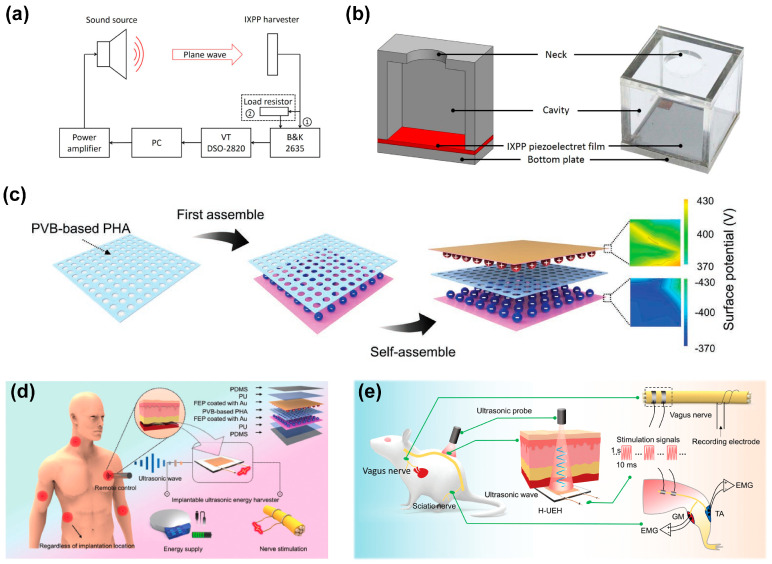
(**a**) Experimental configuration of measurements for output power of IXPP acoustic energy harvesters and (**b**) cross-sectional view and optical image of a low-frequency IXPP film acoustic energy harvester made of a Helmholtz resonator and an IXPP film. Reprinted with permission from Ref. [37]. Copyright 2019, IOP Publishing Ltd. (**c**) Schematic diagrams showing the process of preparing the H-UEH, (**d**) illustration of H-EUH as implantable bioelectronics for energy supply and neuroprosthetics, and (**e**) schematic illustration of the H-UEH driven by ultrasound for stimulating peripheral nerves. Reprinted with permission from Ref. [16]. Copyright 2022, Wiley-VCH Verlag.

IXPP films, due to their low cost, low impedance, flexibility, biocompatibility, and environmental friendliness, are anticipated to play a significant role in the future of acoustic energy harvesting. In 2019, Xue et al. [37] reported on the harvesting of acoustic energy using IXPP films in both ultrasonic and low-frequency ranges. Their experimental design for measuring the output power of IXPP acoustic energy harvesters is illustrated in Figure 10a. With an input Sound Pressure Level (SPL) of 100 dB (or 2 Pa) and a resonance frequency of 53 kHz, an IXPP film harvester yielded a maximum output power of 7.2 nW. To enhance the efficiency of acoustic energy harvesting, they used a Helmholtz resonator (HR) to amplify the incident sound pressure, attaching IXPP films to the resonator’s bottom plate, as demonstrated in Figure 10b. At an input SPL of 100 dB and a resonance frequency of 900 Hz, this IXPP energy harvester could produce a maximum output power of 10.3 nW. The incorporation of an HR can significantly improve the efficiency of FENG’s acoustic energy collection. In 2021, Song et al. [116] conducted comprehensive research on multi-frequency acoustic energy harvesting using differently sized HRs with IXPP films in the 300~800 Hz range. They enhanced the output power by connecting several HRs of the same size in series, achieving low-frequency acoustic energy harvesting. 

FENG films can effectively harvest low-frequency mechanical vibration and sound wave energy, making them suitable for operating most mechanical devices with reduced dependence on conventional batteries [117]. As depicted in Figure 10c, Wan et al. [16] proposed a multi-layer FENG with enhanced strain piezoelectricity by introducing a parallel-connected air hole array (PHA) in the dielectric layer between a pair of electrets for a highly efficient ultrasonic energy harvester (H-EUH). This device, shown schematically as implantable bioelectronics for energy supply and neuroprosthetics in Figure 10d,e, could be driven by ultrasound for peripheral nerve stimulation. When implanted into tissues at a depth of 5~10 mm, the device could generate a peak output power of 13.13 mW and a short-circuit current of 2.2 mA under an ultrasonic probe setup at 25 mW cm^−2^, demonstrating its potential for powering implantable bioelectronic devices and functioning as neuroprosthetics for peripheral nerve stimulation.

## 4. Summary and Perspectives 

This review seeks to highlight the extraordinary potential and varied applications of the FENG within the realm of acoustics. With its unique cellular structure, exceptional flexibility, and high piezoelectric coefficient, FENG can facilitate bidirectional conversion between sound waves and electrical energy. Coupled with its distinctive attributes, such as efficient energy conversion, it becomes a promising tool with extensive potential applications in acoustics. These applications include acoustic sensing, acoustic actuation, and acoustic energy harvesting.

However, despite the promising developments and potential, several challenges must be addressed concerning the application of FENG in acoustics. Primarily, a comprehensive understanding of FENG’s durability and reliability under varying environmental conditions is lacking. It is essential to evaluate FENG’s performance under different temperature, humidity, and pressure conditions, as well as during extended use. This is particularly critical for applications in harsh environments, such as underwater acoustic sensing and outdoor energy harvesting [96]. Secondly, the piezoelectric coefficient of FENG can be impacted by factors like abrasion and mechanical degradation. Thus, optimizing device design to enhance its stability and durability remains a challenge. Thirdly, FENG’s fabrication process includes intricate procedures such as high-pressure gas injection, thermal treatment, and electrical charging [17]. Enhancing this fabrication process to make it more cost-effective and scalable for industrial applications is another critical task. Lastly, a more comprehensive and accurate theoretical model is required to predict FENG’s behavior and performance across various acoustic applications. Such a model would significantly assist in the design and optimization of FENG-based devices. 

Moving forward, the future of FENG in acoustics lies in overcoming these challenges and maximizing its potential. Progress in materials science and fabrication technology could open the way for the development of more efficient, reliable, and scalable FENG devices. Furthermore, the integration of FENG with other technologies such as artificial intelligence and the Internet of Things could lead to innovative applications, ranging from smart acoustic sensors capable of real-time monitoring and analysis to autonomous energy harvesting systems that power our smart devices [118]. The potential of FENG in medical applications also warrants exploration. Beyond ultrasonic imaging, FENG could be utilized in therapeutic applications like targeted drug delivery and non-invasive surgery, capitalizing on its ability to generate focused ultrasound waves [16,32]. With continued research and development, we can anticipate a new era of acoustic technology powered by FENG, revolutionizing our interaction with sound and its applications in our daily lives.

## Figures and Tables

**Table 1:** Application, manufacturing methods, and charging methods of various FENGs.

Application	Materials	Manufacturing Methods	Charging Methods	Piezoelectric Coefficients	Year
Energy harvesting	PVB/FEP	FEP-PVB-FEP layer stack	Corona discharging	4680 pC/N	2022 [16]
Energy harvesting	PET/silk	PET-silk-PET layer stack and hot-pressing	Corona discharging	1600 pC/N	2020 [69]
Energy harvesting	FEP	Hot-pressing	Contact charging	3.0 Vm/N	2018 [68]
Sensing	PP	3D-printing	Contact charging	200 pC/N	2020 [70]
Sensing	P(VDF-TrFE)	3D-printing	Contact charging	1600 pC/N	2022 [71]
Sensing	FEP/PTFE	Patterning and fusion bonding method	Contact charging	400 pC/N	2018 [72]
Sensing/energy harvesting	PVDF/Cd/INH	Solvent casting	Self-polarization	143 pC/N	2020 [73]
Sensing/energy harvesting	PVDF	Freeze casting	Corona charging	264 pC/N	2019 [74]
Sensing/energy harvesting	FEP/ePTFE	FEP-ePTFE-FEP-ePTFE-FEP stack	Corona charging with an extra boost of external positive and negative ions	1600 pC/N	2022 [46]

Abbreviations as follows: PVB, polyvinyl butyral; FEP, fluorinated ethylene propylene; PET, polyethylene terephthalate; P(VDF-TrFE), poly(vinylidene fluoride-trifluoroethylene); PVDF, poly(vinylidene fluoride); PTFE, polytetrafluoroethylene; Cd, Cadmium; INH, isoniazid; ePTFE, expanded polytetrafluoroethylene.

**Table 2:** Common FENG devices for acoustic sensing.

Materials	FEP	PP	PP	FEP/PTFE
Applications	Microphone	Ultrasonic localization	Ultrasonic medical imaging	Nondestructive testing
Year	2014 [84]	2011 [31]	2020 [32]	2018 [72]

**Table 3:** Common FENG devices for acoustic actuation.

Materials	ePTFE	PP	IXPP	PP
Applications	Loudspeaker	Loudspeaker	Acoustic levitator	Vortex generator
Year	2022 [35]	2017 [22]	2020 [36]	2011 [110]

**Table 4:** Common FENG devices for acoustic energy harvesting.

Materials	IXPP	IXPP	PVB/FEP
Applications	Ambient sound energy harvesting	Ambient sound energy harvesting	Implantable electronics power supply
Year	2017 [37]	2021 [116]	2020 [16]
